# Modelos para estimar la variación biológica y la interpretación de resultados seriados: bondades y limitaciones

**DOI:** 10.1515/almed-2020-0017

**Published:** 2020-09-22

**Authors:** Jorge Díaz-Garzón, Pilar Fernández-Calle, Carmen Ricós

**Affiliations:** Comisión de Calidad Analítica, SEQC^ML^ , Barcelona, España; Servicio Análisis Clínicos, Hospital Universitario La Paz, Madrid, España

**Keywords:** diseño estadístico, metodología, variación biológica

## Abstract

La variación biológica (VB) tiene múltiples aplicaciones en diversos campos del laboratorio clínico. Hay dos formas de relacionar el concepto de VB y los modelos estadísticos. Por un lado existen modelos para el cálculo de estimados de VB (intra e inter individual) y por otro, existen modelos que tienen en cuenta la VB y otros factores para la definición de rangos que ayudan a la interpretación de resultados seriados en un mismo individuo. Dentro de los modelos estadísticos dirigidos al cálculo de los estimados de VB existen dos tipos: A. Métodos directos. Estudios prospectivos, diseñados exclusivamente para el cálculo de estimados de VB: i. Modelo clásico: desarrollado por Harris y Fraser, revisado por EFLM-BVWG. ii. Modelos de efectos mixtos iii. Modelo bayesiano. B. Métodos indirectos. Estudios retrospectivos basados en extraer estimados de VB a partir de resultados que provienen de grandes bases de datos. Big-data. Ambos tipos presentan una serie de características que es importante conocer porque pueden condicionar su aplicabilidad en diferentes situaciones o poblaciones. Entre los modelos para definir rangos que ayudan a la interpretación de resultados seriados en un individuo encontramos: A. Valor de referencia del cambio (VRC). B. Red de datos bayesiana. En resumen, esta revisión pretende dar un enfoque general sobre los modelos para definir los componentes de VB así como otros para aplicarlos en el seguimiento de pacientes, que deberían ser explorados en el futuro para personalizar y mejorar la información aportada por el laboratorio clínico, aprovechando al máximo los recursos disponibles.

## Introducción

Desde la primera descripción del concepto de variación biológica (VB) definido por Harris y Fraser a mediados del siglo 20 [1], se han realizado múltiples estudios experimentales y, por tanto, prospectivos, más o menos complejos para conseguir estimados robustos de VB que pudieran servir como datos de referencia, de forma que fueran transferibles a diversas poblaciones, entornos y patologías.

Dentro del concepto de VB es posible distinguir dos componentes, la VB intra- e interindividual. El primero, hace referencia a la fluctuación de la concentración de un mensurando alrededor de su punto homeostático en un individuo, mientras la segunda define la variación entre los puntos homeostáticos de diferentes individuos [1], [2], [3]; ambos estimados se expresan como coeficientes de variación y se describen con las siglas CV_I_ y CV_G_, respectivamente, de acuerdo a la terminología estandarizada recomendada por Simundic y cols [4].

La VB tiene múltiples aplicaciones en diversos campos del laboratorio clínico, como el control interno del proceso analítico, los programas de garantía externa de la calidad, el cálculo del valor de referencia del cambio [1], la verificación y validación de métodos analíticos, el criterio para definir la máxima desviación debido a inestabilidad de las muestras biológicas [5], además de para realizar estudios de interferencias y establecer el límite de cuantificación de un mensurando [6].

Hay dos formas de relacionar el concepto de VB y los modelos estadísticos. Por un lado existen modelos para el cálculo de estimados de VB (intra e inter individual) y por otro, existen modelos que tienen en cuenta la VB y otros factores para la definición de rangos que ayudan a la interpretación de resultados seriados en un mismo individuo.

## Modelos para el cálculo de estimados de VB

Dentro de los modelos estadísticos dirigidos al cálculo de los estimados de VB existen dos tipos:

### A. Métodos directos

Estudios prospectivos, diseñados exclusivamente para el cálculo de estimados de VB:

- Modelo clásico: desarrollado por Harris y Fraser, revisado por EFLM- BVWG.
- Modelos de efectos mixtos.
- Modelo bayesiano.

### B. Métodos indirectos

Estudios retrospectivos de los que se derivan estimados de VB a partir de resultados que provienen de grandes bases de datos. *Big-data.*

Ambos tipos presentan una serie de características que es importante conocer porque pueden condicionar su aplicabilidad en diferentes situaciones o poblaciones.

### A. Métodos directos

#### i. Modelo clásico

Se desarrolló en los años 60, por Fraser y Harris [1] y, desde entonces, la mayoría de los trabajos que presentan estimados de VB se basan en este diseño, siguiéndolo de una manera más o menos estricta. En base a la recopilación de todos ellos, la Comisión de Calidad Analítica de la SEQC^ML^ publicó en 1997 una base de datos que compilaba los estudios existentes y que se ha mantenido actualizada hasta 2014 [7], [8], sirviendo de herramienta de gran utilidad para los profesionales del laboratorio clínico.

El modelo clásico descrito por Fraser y Harris, que requiere la medición por duplicado de las muestras de los sujetos estudiados, asume tres características necesarias e imprescindibles: que los datos de VB sigan una distribución normal, que la distribución de las variables homocedástica (homogeneidad de varianzas en todo el intervalo de concentraciones, la cual implica que la distribución de los resultados es homogénea entre los replicados de una misma muestra y los diferentes sujetos) y que los sujetos no presenten ninguna tendencia a lo largo del estudio (estado estable). El método estadístico recomendado para el cálculo de los estimados de VB consiste en aplicar un análisis de la varianza (ANOVA) anidado tras un análisis exhaustivo en búsqueda de valores extremos (*outliers*) a tres niveles (intra-duplicados, intra-sujeto e inter-sujeto).

Este abordaje se basa en el concepto de que la variación total de las mediciones, expresada como coeficiente de variación (CV_T_), es igual a la suma de las variaciones pre-analítica (CV_PRE_), analítica (CV_A_) y biológica intra-individual (CV_I_). Una vez controlada y conocida la variación analítica y realizando el experimento en condiciones pre-analíticas estandarizadas, esta última fuente de variación puede descartarse, quedando en la ecuación por tanto, la CV_I_, que sería igual a la diferencia entre la variación total y la variación analítica.

$$VT=CV_{PRE}+CV_{A}+CV_{I}\to CV_{I}=CV_{T}-CV_{A}$$

Ahora bien, este modelo presenta una serie de “exigencias” o requisitos para la obtención de estimados de VB robustos. Así pues, es necesario disponer de un adecuado número de sujetos, así como de muestras por sujeto y de replicados. Para ello, se debe tener en cuenta el ratio entre la imprecisión analítica de los métodos de medida y la CV_I_ que se prevé obtener [9], siendo recomendable que sea inferior a 1 y deseable que sea inferior a 0.5 [1].

Recientemente la *European Federation of Laboratory Medicine* (EFLM) a través del *Working Group* de Variación Biológica (EFLM-BVWG) junto con el *Task Group on Biological Variation Databa*se (TG-BVD) ha revisado los estudios disponibles en la bibliografía, a través de la aplicación de una herramienta de lectura crítica diseñada por el grupo. Así, los estudios se han clasificado en cuatro categorías (A, B, C y D) en orden creciente a su nivel de calidad. Además, ha aplicado un metanálisis para estimar los componentes de VB, CV_I_ y CV_G_, así como sus intervalos de confianza al 95% [10]. Con ellos se ha elaborado una nueva base de datos de VB publicada en la página web de EFLM [11] en mayo de 2019. La mayoría de los estudios publicados e incluidos en la nueva base de datos se adaptan a este diseño clásico, sin embargo, muchos obtienen los estimados por simple sustracción de varianzas, es decir, restan directamente la variación analítica a la variación total observada para obtener los estimados de CV_I_, en lugar de utilizar otro método estadístico más adecuado como el ANOVA, haciendo que su calidad metodológica sea menor. Además, presentan otras debilidades relacionadas con la asunción de estabilidad de los sujetos estudiados, el método para obtener el CV_A,_ la homocedasticidad de las varianzas, así como con el tratamiento para la eliminación de *outliers* [10], [12].

La ventaja más importante de este método es que está ampliamente probado y contrastado, y por tanto es muy utilizado y conocido. Es un método prospectivo y controla las variables pre-analíticas de manera que aseguramos unos estimados veraces.

Sin embargo, presenta algunas limitaciones como son:

- No existencia de homoscedasticidad de los sujetos incluidos para algunos mensurandos.
- *Outliers* eliminados con excesiva rigurosidad que podrían hacer que se pierda transferibilidad y potencia estadística. Esto es debido a que el tratamiento de *outliers* es manual pese a seguir ciertos criterios recomendados (Reed, Cochrane, etc.) y cabe la posibilidad de que se estén eliminando resultados valiosos, representativos de los sujetos.
- La mayoría de los estudios incluyen un número reducido de sujetos debido a que aumentar el tamaño muestral encarece el presupuesto y dificulta la realización del estudio. Además, el número de muestras obtenidas por sujeto (visitas) no suele ser elevado porque compromete la adherencia al estudio de los participantes.
- Existencia de tendencias significativas que afectan a la estabilidad del estado de equilibrio (homeostasis) en el tiempo asumido para los sujetos estudiados y que comprometen la obtención de estimados veraces. Si afectan a un solo sujeto, existe la posibilidad de eliminar el sujeto en el que se detecta la tendencia. Por otro lado, cuando se detectan tendencias que afectan a todos los resultados, como por ejemplo, las variaciones estacionales, se pueden realizar correcciones para minimizar ese factor. Lamentablemente, no existen recomendaciones ni hay un criterio claro y definido en la literatura para solucionar esta problemática.
- Posible existencia de variables no controladas por el investigador que potencialmente podrían estar sesgando los estimados obtenidos.

#### ii. Modelos de efectos mixtos

La base matemática de este modelo es la misma que la del modelo clásico debido a que el método estadístico utilizado es un ANOVA anidado, sin embargo, la estimación se realiza a través de un modelo mixto, que tiene en cuenta otras variables distintas a la regulación homeostática. En este modelo, también es necesario que la distribución de los resultados sea normal y las varianzas homogéneas para conseguir la obtención de estimados fiables de VB [13]. La diferencia más importante con respecto al método clásico, es que se pueden recoger variables que *a priori* pueden considerarse potencialmente influyentes sobre la VB de las magnitudes estudiadas. Después, estas variables pueden incluirse en el modelo y medir la magnitud del efecto (influencia) que tienen sobre la VB.

En general, este modelo incluye dos tipos de efectos: los efectos fijos, que son las variables con potencial influencia (edad, sexo, medicación, estado de salud, etc.) y los efectos aleatorios, que son los estimados de VB e imprecisión (CV_A_, CV_I_ y CV_G_).

La ventaja principal de este modelo radica en que permite conocer si existe influencia de alguna variable sobre la VB y además cuantificar su efecto. Por otra parte, si se han recogido previamente las variables que potencialmente afectan a la VB, el tratamiento de *outliers* no necesita ser tan exhaustivo [13].

Los inconvenientes principales son los anteriormente descritos para el modelo clásico y se añade la necesidad de tener conocimiento previo de las variables potencialmente influyentes, además, el método estadístico es más complejo y requiere conocimientos matemáticos más profundos. En este modelo, para incorporar de manera simultánea diferentes variables, necesitamos tamaños muestrales muy grandes. Por último, un inconveniente importante es que este tipo de modelo no está completamente validado en diferentes estudios de VB [13].

#### iii. Modelo bayesiano

Este modelo se ha desarrollado y publicado recientemente por Roraas y cols. [14] y utiliza un método bayesiano para el cual no es imprescindible que las varianzas sean homogéneas. Esto hace que se pueda evitar realizar un tratamiento de *outliers* tan estricto que nos podría hacer perder datos potencialmente valiosos para la estimación de la VB [9].

El hecho de que no requiera un análisis de *outliers* a tres niveles, ni cumplir criterios de homocedasticidad, hace que la estimación de los componentes de la VB sea más simple, menos manual y por tanto más fiable y lo que aún es más importante, estandarizable. Por otra parte, este método permite el cálculo individualizado del CV_I_ para cada sujeto, pudiendo dar una estimación con percentiles (mediana y rango intercuartílico de CV_I_ y CV_G_), en lugar de un valor central como hacen otros modelos (media).

Este modelo ha sido verificado frente a los métodos tradicionales, habiéndose demostrado que estos modelos obtienen estimados similares de VB. Para realizar estas comparaciones Roraas y cols. [14] utilizan los datos resultantes del estudio EuBIVAS para el cloro y los triglicéridos [15]. Es decir, este modelo ya ha sido validado con datos reales de estudios robustos de VB, lo que supone una garantía para este tipo de abordajes.

Sin embargo, la principal desventaja de este modelo es el nivel de conocimientos matemáticos y de programación necesarios en *softwares* estadísticos que permiten su aplicación. Otra desventaja adicional es la necesidad de estudios previos de VB para incluir sus estimados como información *a priori* (hiperparámetros), de forma que si la información *a priori* que se introduce en el modelo es incorrecta se pueden obtener conclusiones incoherentes o incompatibles con las hipótesis definidas en el diseño del estudio.

En resumen, este tipo de modelo se debería utilizar siempre que se tenga apoyo estadístico y es muy recomendable cuando se estudie la VB en magnitudes no homocedásticas en las que se obtiene un elevado porcentaje de valores atípicos no explicable por la información registrada durante el estudio.

### B. Métodos indirectos. *Big data*

Es un modelo en auge en la actualidad, pero aún cuenta con pocos estudios realizados y sin consenso de cuál es el diseño más apropiado (método estadístico, criterios de inclusión, etc.).

Esta estrategia asume que la mayoría de los estimados no están afectados por el estado de salud de los sujetos, ya que el análisis de valores atípicos elimina los resultados patológicos [16]. Pueden considerarse como ejemplos los estudios de Loh y cols. en población pediátrica que acude a la atención primaria, con resultados de CVI similares a los obtenidos en adultos sanos [17], [18].

Las ventajas más importantes son que se pueden estudiar diferencias debidas a la edad, sexo de los sujetos, a la duración del estudio [16], [17], [18] incluso diferentes patologías [19]. Como incluye un número mayor de sujetos con respecto a los estudios prospectivos (pueden ser varios miles, porque se parte de los datos del sistema informático del laboratorio), tiene mayor transferibilidad y potencia estadística. Supone un bajo coste económico porque consume pocos recursos humanos y materiales y no requieren fase experimental.

Entre las desventajas cabe destacar que las variables pre-analíticas no están estandarizadas, se desconoce si los sujetos estudiados están en estado de equilibrio homeostático, el CV_A_ proviene del control interno del proceso analítico (muestras control estabilizadas con distinta matriz que las muestras de los pacientes y diferentes concentraciones que las muestras de los sujetos incluidos en el estudio) y no obtenido a partir de duplicados de muestras de los sujetos estudiados, que a menudo estos estudios tienen un bajo número de muestras por sujeto, e intervalos de muestreo variables que, en el caso de algunas magnitudes, podrían limitar la robustez de los resultados. Algunos de estos estudios multicéntricos, incluyen resultados obtenidos con diferentes métodos de medida y lotes de reactivo.

## Modelos para definir rangos que ayudan a la interpretación de resultados seriados en un individuo

- Valor de referencia del cambio (VRC)
- Red de datos bayesiana

### A. Valor de referencia del cambio (VRC)

El valor de referencia del cambio define un rango a partir del cual la diferencia entre dos resultados seriados de un individuo podría considerarse biológicamente significativa. El VRC se calcula a partir del error analítico del método de medida y la variación de la magnitud a estudio en el sujeto y para ello incluye el CV_A_ del laboratorio y el CV_I_ respectivamente [20]. Este concepto asume que todos los individuos tienen el mismo CV_I,_ y por tanto requiere estimados de CV_**I**_ robustos y representativos de la población sobre la que se aplica este concepto para su seguimiento. La fórmula clásica para el cálculo del VRC es la siguiente, con probabilidad Z de 1,96 y 1,65 para cambios bi- y uni-direccionales, respectivamente [1]:

$$VRC=2^{1/2}Z{\left(CV_{A}^{2}+CV_{I}^{2}\right)}^{1/2}$$

A diferencia del concepto clásico, que considera que todas las magnitudes siguen una distribución normal y el rango es simétrico, el método actualmente recomendado para el cálculo del VRC se basa en un método logarítmico que define un rango asimétrico [21]. La fórmula es:

$$\begin{array}{c} VRC_{pos}=\left(\exp\left(1,962^{1/2}\alpha \right)-1\right)100\\ VRC_{neg}=-\left(\exp\left(1,962^{1/2}\alpha \right)-1\right)100\\ \alpha ={\left(Ln\left(CV_{T}+1\right)\right)}^{1/2};\;CV_{T}=CV_{A}+CV_{I} \end{array}$$

Las magnitudes susceptibles de su interpretación mediante este método son las que están sometidas a una mayor regulación homeostática, es decir aquellas magnitudes con un bajo Índice de individualidad (II<0,6) como es el caso de la creatinina, los electrolitos y algunas magnitudes o parámetros hematológicos. Este índice relaciona los estimados de VB intra e interindividuales (II=CV_I_/CV_G_) [1]. Los resultados seriados de las magnitudes con elevada individualidad deberían ser interpretados mediante el VRC como alternativa a los intervalos de referencia biológicos.

Las principales limitaciones de este concepto son su desconocimiento por parte de la mayoría de los clínicos y que los sistemas informáticos de laboratorio no están plenamente adaptados para su aplicación.

Otro de los aspectos a tener en cuenta es que la mayoría de los estudios de VB han estandarizado las condiciones preanalíticas para evitar la influencia y el sesgo a la hora de obtener los estimados de VB. Sin embargo, en un entorno real, estas variables no siempre están estandarizadas por los laboratorios y esta fuente de variación no se tiene en cuenta cuando definimos el rango. En este contexto, el VRC podría ser demasiado estricto y los cambios entre resultados interpretados como biológicamente significativos cuando no lo son.

Por otra parte, el CV_A_ incluido en la ecuación procede del control interno, lo que implica que en algunas magnitudes en las que el CV_A_ es diferente en función de la concentración del mensurando, debería aplicarse un CV_A_ distinto para cada concentración, dependiendo de los resultados obtenidos y esto dificulta aún más su aplicación.

### B. Red de datos bayesiana

Los modelos bayesianos permiten ir actualizando, a la vez que adecuando la probabilidad de obtener un resultado en un rango utilizando información previa. Estos modelos son flexibles y mejoran progresivamente a medida que integramos información. En el momento basal, la información previa puede obtenerse de artículos previamente publicados o de fuentes fiables [11].

Estos modelos han sido desarrollados por Sottas y cols. [22] en el mundo de la medicina de laboratorio y aplicados por la Agencia mundial antidopaje (AMA) para la detección del consumo de sustancias ilícitas. El método empleado para el seguimiento de los atletas se denomina pasaporte biológico y en lugar de detectar la sustancia ilícita en el organismo, monitoriza marcadores indirectos de su consumo (hemoglobina, reticulocitos, etc.).

Para predecir el intervalo específico en que una magnitud concreta varía en un individuo determinado hay que tener en cuenta tanto información previa basada en la distribución de resultados de la población específica a la que pertenece (edad, sexo, raza, patología) como los resultados del propio individuo [23].

Este modelo se apoya en una red bayesiana jerárquica, constituida por varios niveles, que se construye a partir de resultados de pacientes y variables de heterogeneidad como sexo, edad y otros factores. En un primer nivel, separa los datos en diferentes distribuciones (grupos) en función de estas variables de heterogeneidad. En un segundo nivel, utiliza como información *a priori* (hiperparámetros) los estimados de BV derivados de estos grupos [24].

Posteriormente, tras incorporar en el modelo resultados previos del propio individuo se obtienen distribuciones que definen un rango que predice el resultado del individuo con un grado de robustez superior al de los anteriores niveles.

En resumen, este modelo define intervalos de referencia específicos para un individuo, utilizando información previa, es decir, parte de distribuciones preliminares e incorpora datos que dan información al modelo y derivan en distribuciones posteriores más robustas. Además, si cuenta con suficientes resultados incluidos en la base de datos de la población que va a estudiar, no necesita estudios previos específicos de VB (hiperparámetros).

En los ensayos clínicos en los que se ha utilizado este modelo se ha observado que el número de pacientes o muestras a tener en cuenta para satisfacer los requisitos del ensayo podrían decrecer de manera significativa o el ensayo podría finalizar con anterioridad. Por ejemplo, Sottas indica que si se usan los intervalos de referencia poblacionales se necesitan 600 sujetos para detectar un cambio en la concentración de creatinina en suero de 0,06 mg/dL, si se utiliza un análisis de covarianza con respecto a los valores basales se requieren 210, mientras que si se emplea el análisis bayesiano son necesarios 20 individuos. Es decir que este modelo podría, trasladado a la práctica clínica, ser una herramienta muy útil para ayudar a la interpretación de resultados de laboratorio [24].

La red de datos bayesiana asume que no existe error analítico, para conseguir esto, todos los resultados deben ser obtenidos con el mismo procedimiento analítico, asegurando que no existe sesgo y que la imprecisión es baja y está absolutamente controlada. Esta circunstancia hoy en día no se cumple para muchas de las magnitudes utilizadas a diario en el laboratorio para el seguimiento de muchas patologías. Quizá, en un futuro, se podrían incorporar en este modelo los indicadores del error analítico obtenidos a través del control de calidad interno por el laboratorio como hiperparámetros y así predecir con mayor robustez los intervalos indicativos de cambio.

Otra desventaja importante es que estas bases de datos no son públicas y no existe acceso a ellas, por lo que todavía no son aplicables en la práctica en los laboratorios, al menos, de manera gratuita.

En resumen, esta revisión pretende dar un enfoque general sobre los modelos para definir los componentes de VB así como otros para aplicarlos en el seguimiento de pacientes, que deberían ser explorados en el futuro para personalizar y mejorar la información aportada por el laboratorio clínico, aprovechando al máximo los recursos disponibles.
